# Some Considerations about the Anodic Limit of Ionic Liquids Obtained by Means of DFT Calculations

**DOI:** 10.3390/e25050793

**Published:** 2023-05-12

**Authors:** Annalisa Paolone, Simone Di Muzio, Oriele Palumbo, Sergio Brutti

**Affiliations:** 1 Consiglio Nazionale delle Ricerche, Istituto dei Sistemi Complessi, Piazzale Aldo Moro 5, 00185 Rome, Italy; simone.dimuzio1@graduate.univaq.it (S.D.M.); oriele.palumbo@roma1.infn.it (O.P.); sergio.brutti@uniroma1.it (S.B.); 2 Department of Physical and Chemical Sciences, University of L’Aquila, Via Vetoio, 67100 L’Aquila, Italy; 3 Department of Chemistry, Sapienza University of Rome, Piazzale Aldo Moro 5, 00185 Rome, Italy

**Keywords:** QSAR, machine learning, DFT calculations, anodic stability, ionic liquids

## Abstract

Ionic liquids are good candidates as the main component of safe electrolytes for high-energy lithium-ion batteries. The identification of a reliable algorithm to estimate the electrochemical stability of ionic liquids can greatly speed up the discovery of suitable anions able to sustain high potentials. In this work, we critically assess the linear dependence of the anodic limit from the HOMO level of 27 anions, whose performances have been experimentally investigated in the previous literature. A limited *r* Pearson’s value of ≈0.7 is found even with the most computationally demanding DFT functionals. A different model considering vertical transitions in a vacuum between the charged state and the neutral molecule is also exploited. In this case, the best-performing functional (M08-HX) provides a Mean Squared Error (MSE) of 1.61 V^2^ on the 27 anions here considered. The ions which give the largest deviations are those with a large value of the solvation energy, and therefore, an empirical model that linearly combines the anodic limit calculated by vertical transitions in a vacuum and in a medium with a weight dependent on the solvation energy is proposed for the first time. This empirical method can decrease the MSE to 1.29 V^2^ but still provides an *r* Pearson’s value of ≈0.72.

## 1. Introduction

Batteries are nowadays one of the most versatile energy storage systems for portable electronics and are also reaching high spread for fueling electric vehicles. Especially for the latter application, there is a need for high-energy devices. Among the possible strategies to achieve this goal, the formulation of batteries working at higher voltages (approaching 5V) is extremely attractive, but this choice poses demanding challenges on the constituent materials of the cells [1]. One of the problems is the anodic instability of the common electrolytes for Li-ion batteries. The typical aprotic electrolyte formulations are based on mixtures of organic carbonates, and on paper, they are stable up to 4.5 V vs. Li. At higher potentials, these electrolytes can decompose, producing degradation products which can damage or hinder the correct functioning of any batteries [1]. Among other options to improve the electrochemical stability of electrolytes, the use of ionic liquids, as additives, co-solvents or solvents, has been proven to disclose remarkable enhancement in the electrochemical stability window of electrolytes [2,3,4,5]. Ionic liquids have many physicochemical properties which make them ideal candidates to replace organic carbonates as electrolytes in electrochemical devices: high thermal stability, low volatility, large liquid range, large electrochemical stability and high ionic conductivity [2,3,4,5].

Experimentally, the electrochemical stability of ionic liquids has been investigated in many systems, and it is clear that the fluorination of the anions composing the ILs is a key ingredient to obtain electrolytes for high potential application [2,3,4,5]. Overall, the identification of novel ILs would benefit from fast screening methods to find molecular pairs with good electrochemical performances, without addressing expensive trial-and-error procedures, which are extremely time-consuming. 

Any electrolyte is electrochemically stable in the potential range, which avoids the irreversible reduction or oxidation of salts and solvents’ constituents. Being ionic liquids formed by charged species, there is a general agreement that the irreversible oxidation (removal of one electron) occurs mostly from the anion, while the irreversible reduction (addition of one electron) takes place on the cation. Oxidations and reductions occur through transitions between states at different energy levels: these estimates allow us to evaluate the potential difference limiting the electrochemical stability. This concept is quite simple, but the choice of the method to calculate the energy difference between the states for oxidations or reductions is not trivial, and many options have been proposed in the literature.

The first attempt to calculate the highest voltage at which ILs could be used assumed a direct correlation with the energy of the highest unoccupied molecular orbital (HOMO) of anions [6,7]. A second model correlated the anodic limit to the energy difference for a vertical transition from the charged ion to the neutral state, either in the gas phase or in the presence of a suitable polarizable medium (PM) [8,9]. With the inclusion of a polarizable medium, the anodic limit is always highly overestimated [8], while better results are obtained with calculations in a vacuum. Similar DFT calculations were also performed on cations to determine their cathodic limit [10]. Numerous DFT functionals were exploited to calculate the electrochemical stability windows of many ionic liquids, using either vertical or adiabatic transitions [11,12,13]. More recently, some authors introduced DFT calculations based on thermodynamic cycles also involving the solvation of the single ions [14,15]. High-throughput screening of hundreds of possible functionalized anions and cations was reported by Cheng [14], using DFT calculations with the B3LYP functional. Besides DFT methods, in the literature, some more complex calculations of the electrochemical stability of ionic liquids based on a combination of DFT and molecular dynamics calculations [16] or ab-initio molecular dynamics [17] are available. The problem of finding an effective way to estimate the electrochemical stability of ionic liquids continues to fascinate researchers, and some recent publications were devoted to this theme [18,19,20,21].

In our previous works, we explored different methods to calculate the anodic limit of ionic liquids, previously proposed in the literature, all based on ab-initio or DFT calculations [22,23]. For an oxidation reaction where the reagent (initial state) is the chemical specie *A* with net charge *n*, the energy involved in the oxidation reaction, $A^{n}\to A^{n+1}+e^{-}$, is

(1) $$\Delta E_{anodic}=E_{tot}\left(A^{n+1}\right)-E_{tot}\left(A^{n}\right)$$

and the anodic limit is

(2) $$Anodic\;limit\;\left(V\;vs.\;Li\right)=\frac{\Delta E_{anodic}}{F}-1.46$$

where *F* is the Faraday constant, and the term −1.46 V is used to refer these limits to the standard Li^+^/Li^0^ [11].

We displayed that performing this kind of calculation on ionic couples gave unphysical results, as similar values of the anodic limit were obtained for ionic liquids containing either the TFSI^−^ or the Cl^−^ anions, which experimentally differ by at least 2 eV [24]. On the contrary, much better results were obtained considering the single anions [22]. The comparison with the experimental data reported in Ref. [25] for ILs containing the TFSI or FSI anion provided evidence that the best way to calculate the anodic limit is by means of a vertical electronic transition (no geometry variation) from the anion to the neutral species [22]. A series of different DFT functionals with increasing levels of complexity, from the Generalized Gradient Approximation to the Range-Separated Hybrid meta-Generalized Gradient Approximation, were compared [23]. The best match with the experimental data reported in Ref. [25] was found by means of the BMK, ωB97M-V and MN12-SX; acceptable results could be obtained by M06-2X, M11, M08-HX and M11-L [23]. Reasonable values were also calculated by means of the less computationally expensive functionals CAM-B3LYP and ωB97X-D [23].

In the present work, we want to extend the investigation by the method defined in our previous papers to other anions to possibly find some best-performing DFT functions, which could be used in the future also in view of predictions on new anions. Moreover, we wanted to explore the possibility to correlate QSAR descriptors to the anodic limit of known anions. In the end, we propose an empirical model which combines the anodic limit calculated in a vacuum with that obtained in a polarizable medium, using a weighting dependent on the solvation energy of the anions.

## 2. Materials and Methods

All calculations reported in the present paper were performed by means of the Spartan20 software [26]. All structures were optimized at the various levels of theory used in the paper, and only the lowest energy structure was considered. In all cases, the 6-31G** basis set was employed, as in our previous papers [22,23]. The QSAR descriptors were calculated, as well as the anodic limit of 27 anions using Equations (1) and (2). In the first part of the paper, all calculations were performed in a vacuum considering vertical transitions, following the results of our previous papers [22,23]. However, we extended the calculation also to vertical transition in a polar solvent (exploiting the solvent 2pentanone, which has an $\varepsilon_{r}$ of 15.2, a value typical for ILs, see Section 3.3) using the Polarizable Continuum Model (C-PCM) algorithm and recalculating the anodic limit using Equations (1) and (2) applied to the values obtained in the medium. The solvation energy used for the development of the empirical model, SE, of the anions was obtained as the electronic energy difference between the anion in the solvent and the anion in a vacuum. The vibrational contribution was omitted, for simplicity, in view of the close resemblance of the vibrations in the two environments. 

**Figure 1:**
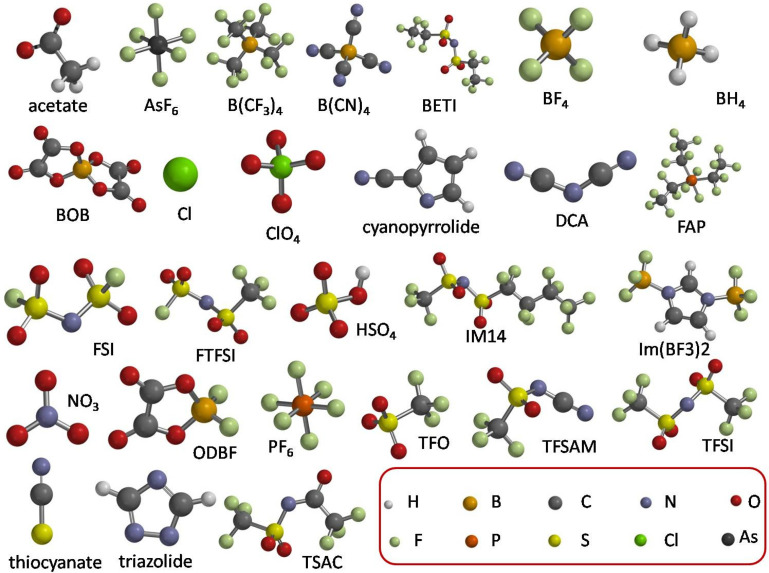
Structure of all anions investigated in this work and legend of the colors of atoms.

Figure 1 reports the structures of the 27 anions whose anodic limits were experimentally studied in the literature, which will be computationally investigated in the present paper. The experimental values are reported in Table 1, together with the proper literature sources. For the comparison with the experimental data, only the papers with a clear reference electrode were selected. In some cases, we could find only one report of the anodic stability of certain anions. On the contrary, for some of them, there is a large literature (see for example TFSI). We considered the mean of the experimental values when they were available. A typical uncertainty of 0.5 V was obtained. For this reason, we considered this uncertainty in the experimental data.

## 3. Results and Discussion

In the following, the computational studies will be rationalized starting from simple QSAR considerations, reported in Section 3.1, proceeding with DFT and ab-initio calculations with different functionals and in different media (Section 3.2) and completing with the proposition of an empirical model relying on calculations in a vacuum and in a dielectric environment, combined with the solvation energy of the anion, that can provide a better agreement with the experimental data than the calculations in a single environment (Section 3.3).

### 3.1. QSAR and Anodic Limit of Different Anions

The QSAR descriptors of the 27 anions were calculated at the MP2, HF, B3LYP, MN-12SX and M11 level of theory, using in all cases the 6-31G** basis set. They include the energy, the HOMO and LUMO levels, polarizability, dipole moment, number of hydrogen-bond donor and acceptor sites, the area, volume, polar surface area, ovality, the accessible area, the polar area and accessible polar area, the minimum and maximum values of the electrostatic potential and the minimum value of the local ionization potential. The used theory levels were chosen as representative of different grades of approximation: MP2 is the Møller–Plesset second-order perturbation theory approximation, HF is the Hartree–Fock theory, B3LYP is a DFT functional based on the simple Global Hybrid Generalized Gradient Approximation (GH-GGA), while both MN-12SX and M11 are classified as the most complex type of DFT functionals (Range-Separated Hybrid meta-Generalized Gradient Approximation (RSH-mGGA)).

As expected, most descriptors, for all types of theory, did not show any correlation with the anodic limit of the different anions. In Figure S1 of the Supporting Information, the dependence of the experimental anodic limit versus the electronic properties of the anions (the energy, the LUMO and HOMO levels and the minimum of the ionization potential (Min_ion-p_)) are reported when calculated at the HF level of theory. Similar results are obtained for the other levels of theory. A correlation can be found in the case of the HOMO level and the minimum of the ionization potential, with a Pearson’s r value for a linear fit of 0.71 and 0.67, respectively (see Figure S1). However, if one tries a linear fit using two independent variables (HOMO level and Min_ion-p_), Min_ion-p_ is excluded from the fit on a statistical basis. Therefore, only the HOMO level has a significant correlation with the experimental anodic limit, even though it is relatively low.

**Figure 2:**
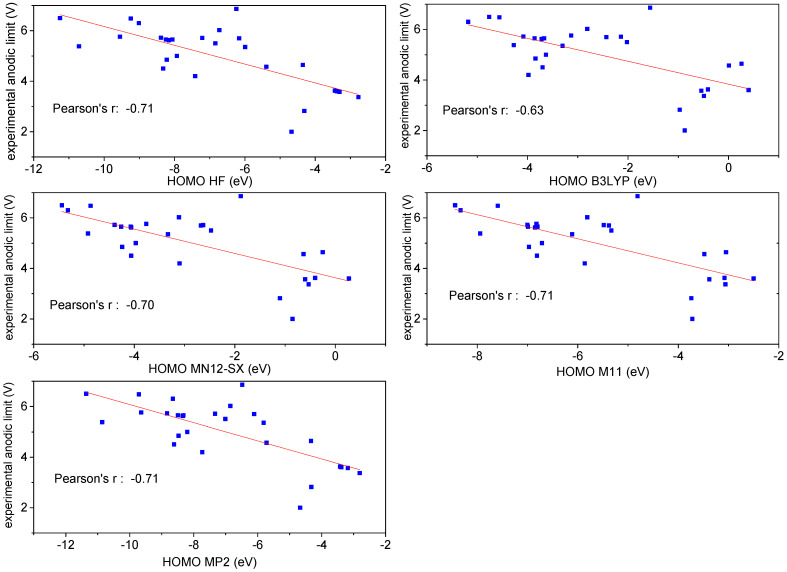
Experimental anodic limit versus the HOMO level calculated by the five different theories and best-fit lines; all calculations were performed using the 6-31G** basis set.

Table 1 displays the values of the HOMO levels calculated by means of the five levels of theory (MP2, HF, B3LYP, MN12-SX and M11), while Figure 2 shows the dependence of the experimental anodic limit from the HOMO level at the different theory levels, together with the best linear-fit lines. The Pearson’s r value is similar for all levels of theory (0.70–0.71), except for B3LYP, which has r≈0.63. Even with the best-performing models, one obtains that only $r^{2}\approx 50\%$ of the variation of the anodic limit of anions depends on the HOMO level. Therefore, the correlation values seem relatively small to be highly predictive for the search of better-performing anions. 

A correlation between the anodic limit and the HOMO level was evidenced many years ago for a limited number of anions (no more than 10) [8,9], and in some cases, at the very beginning of the theoretical investigation of the anodic limit of anions, this correlation was taken for granted [8,9]. Here, we use a more quantitative approach and the availability of experimental studies on a larger number of anions to investigate this possible correlation. However, as reported above, there is a limited correlation between HOMO level and anodic limit of ILs, and, therefore, we moved to the investigation of the anodic limit by DFT calculation based on the electronic properties of the ions.

### 3.2. Ab-Initio and DFT Calculation of the Anodic Limit

The anodic limit of the 27 anions was calculated by means of Equations (1) and (2) using different DFT functionals with different levels of complexity in a vacuum: B3LYP (Global Hybrid Generalized Gradient Approximation, GH-GGA), ωB87X-D and CAM-B3LYP (Range-Separated Hybrid Generalized Gradient Approximation, RSH-GGA), M11-L and BMK (meta-Generalized Gradient Approximation, mGGA), M06-2X and M08-HX (Global Hybrid meta-Generalized Gradient Approximation, GH-mGGA), MN-12SX, M11, ωB87M-V (Range-Separated Hybrid meta-Generalized Gradient Approximation, RSH-mGGA). For comparison, the MP2 theory was also applied. In all cases, the 6-31G** basis set was used. Vertical transitions between the anion and the neutral species were considered [22].

**Figure 3:**
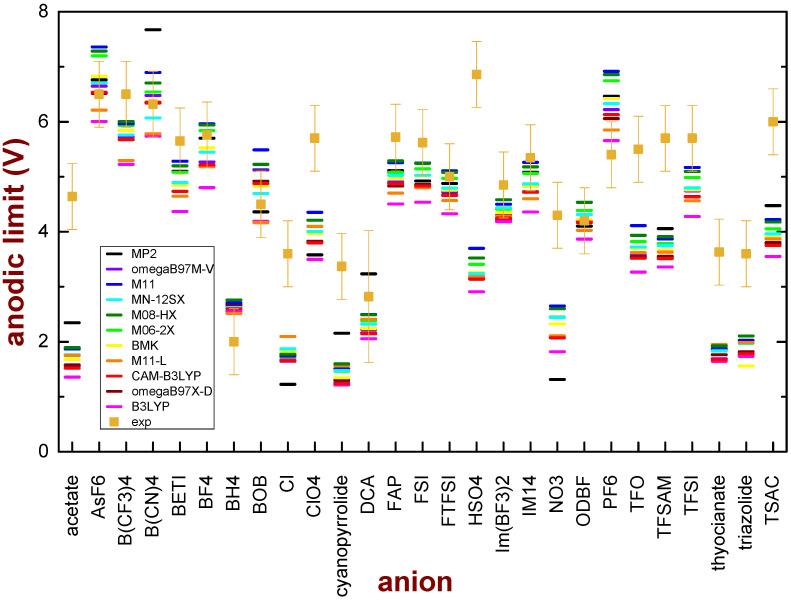
Comparison of the experimental anodic limits of anions and the values obtained by calculations performed on single ions with different DFT functional or the MP2 theory in a vacuum with a vertical transition. In all cases, the 6-31G** basis set was used.

Figure 3 shows the comparison between the experimental and the calculated values of the anodic limit. One can note, in general, that the functionals with the lowest level of approximation, such as B3LYP, give the lowest values of the anodic limit; functionals with a higher level of approximation tend to increase the value of the anodic limit, even though there is not a well-defined trend, in the sense that the highest-level approximations do not always give the highest values of the anodic limit.

A statistical analysis was performed to determine which functional provides the best agreement with the experimental data. The Mean Squared Error (MSE) between the observed experimental data and the values obtained by each functional was calculated and their comparison is reported in Table 2.

The MSE increases in the order M08-HX < M11 < M06-2X < MN-12SX < MP2 < M11-L < BMK < ωB97X-D < ωB97M-V < CAM-B3LYP < B3LYP, passing from 1.61V^2^ for M08-HX to 2.78 V^2^ for B3LYP. Except for ωB97M-V, the agreement between the experimental and computed anodic limit values improves as the level of theory approximation increases.

The values of the MSE are relatively high. It must be noted (see Figure 3), however, that the major contributions to these high figures come from several ions (the same ions for all kinds of functionals): acetate, Cl, ClO_4_, cyanopyrrolide, NO_3_, TFO, TFSAM, thyocianate, triazolide and TSAC. The other 17 anions are quite well described by the proposed model. 

### 3.3. Development of an Empirical Model

**Figure 4:**
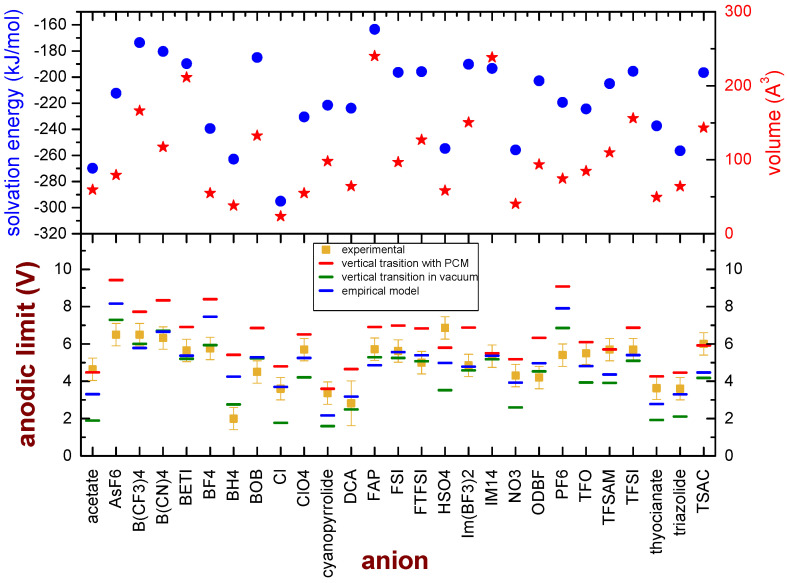
Bottom panel: Comparison of the experimental anodic limits of anions (dark-yellow markers) and the values obtained by calculations performed on single ions by the M08-HX, considering vertical transition in a vacuum (green marks) or vertical transitions in a medium (red marks). Moreover, the best values obtained by means of the proposed empirical model are reported as blue marks. Upper panel: Calculated volume (red stars) and solvation energy (blue dots) of the ions at the M08-HX/6-31G** level of theory.

Since some specific ions clearly deviate from the model based on vertical transitions in a vacuum, one may wonder whether these ions share some common chemical or physical features which can explain such discrepancies. All these ions are quite small; moreover, the model is based on electronic transitions in a vacuum, which is certainly a rough approximation of the real environment in which the oxidation of the anion takes place. We considered the DFT functional which overall gives the best agreement with the experimental data (M08-HX), and we calculated the volume of the isolated ions and the solvation energy, obtained as the difference between the energies of the anions in a dielectric medium (2-pentanone, ε_r_ = 15.2) and in a vacuum. This dielectric constant was chosen as representative of ILs, as they show ε_r_ around 15 [48,49,50]. Moreover, the anodic limit of the 27 anions was calculated considering a vertical transition between the initial anion and the neutral state in the medium. The results are reported in Figure 4. Some general considerations can be derived from this graph: the values of the anodic limit derived from vertical transitions in a polarizable medium are higher than those obtained from vertical transitions in a vacuum, and they are constantly higher than the experimental values. The volume of the anions does not show a clear correlation with the deviation of the anodic limit from the experimental values, as already pointed out by the QSAR analysis. It was reported that a strong correlation between the solvation energies of metal ions and their ionic radii exists, in which the ion solvation energy becomes more positive as the ionic radius increases [51]; however, in the case of the presently investigated anions, for example, not all the ions with a more positive value of the salvation energy are quite large.

On the other hand, most of the 10 anions with the larger deviations from the experimental values (acetate, Cl, ClO_4_, cyanopyrrolide, NO_3_, TFO, TFSAM, thyocianate, triazolide and TSAC) display large values of the solvation energy. Keeping these findings in mind, we explored the possibility to express the anodic limit of anions (*EAL*) by means of an empirical model which linearly combines the anodic limit calculated by vertical transitions in a vacuum ($\Delta E_{vert,vacuum}$) and in a medium ($\Delta E_{vert,medium}$) with a weight dependent on the solvation energy (SE):

(3) $$EAL=\Delta E_{vert,vacuum}+a\left(SE-SE_{0}\right)\Delta E_{vert,medium}$$

where a is a proportionality constant and $SE_{0}$ is a reference value. Both a (−0.003125 V mol/kJ) and $SE_{0}$(−185 kJ/mol) were obtained using a fit procedure. In the lower panel of Figure 4, the EAL values are compared to the experimental values and the figures obtained by vertical transitions in a vacuum. EAL values are closer than $\Delta E_{vert,vacuum}$ to the experimental figures, and indeed the MSE decreases to 1.29 V^2^ and the computed and experimental values differ by less than 1 V, except for five anions (AsF_6_^−^, BF_4_^−^, BH_4_^−^, HSO_4_^−^ and PF_6_^−^). The Pearson’s r for correlation between the calculated anodic limit with the empirical model and the experimental values is 0.72, as reported in Figure S2 of the Supporting Information. The origin of this discrepancy is not straightforward and cannot be easily understood. In fact, there is not apparently a common chemical or physical feature that can easily group together these anions. Overall, these five deviations with respect to the semi-empirical model affect small ions (BF_4_^−^, BH_4_^−^) as well as large anions (AsF_6_^−^, HSO_4_^−^ and PF_6_^−^), the bisulphate anion(which contains a hydrolysable proton as well as sully H-free anions), F-rich anions as well as F-free ions. The only weak common feature of these anions is their strong reactivity towards water that easily leads to bond cleavages (As-F, B-F, B-H, O-H, P-F). Further work is surely needed to shed light on this specific point, and it is already in progress in our laboratory.

## 4. Conclusions

In this paper, a systematic search for reliable algorithms to calculate the anodic limit of ionic liquids was performed. The selected ionic liquids were those for which experimental values to be used as a reference were already available. As a starting point, the relationship between the anodic limit and the HOMO levels of the anions is critically investigated. There is a general linear trend between these two quantities which, however, has a limited *r* Pearson’s value of ≈0.7. On the other hand, DFT calculations of the anodic limit seem more reliable, when considering vertical transitions in a vacuum between the charged state and the neutral molecule. However, even with the best-performing functional (M08-HX) a Mean Squared Error of 1.61 V^2^ is obtained on the 27 anions here considered. The ions which give the largest errors are those with a large value of the solvation energy, and therefore, an empirical model that linearly combines the anodic limit calculated by vertical transitions in a vacuum and in a medium with a weight dependent on the solvation energy is proposed for the first time. This empirical method can decrease the MSE to 1.29 V^2^ and provide an *r* Pearson’s value of ≈0.72.

## Figures and Tables

**Table 1:** Energy of the HOMO level of different anions calculated by MP2, HF, B3LYP, MN12-SX and M11 theories and comparison with the experimental values of their anodic limit. All calculations were performed using the 6-31G** basis set.

	MP2 eV	HF eV	B3LYP eV	MN-12SX eV	M11 eV	Experimental Anodic Limit V
acetate	−4.33	−4.35	0.26	−0.25	−3.05	4.64 [24,27]
AsF_6_	−11.36	−11.25	−4.76	−5.44	−8.44	6.5 [7]
B(CF_3_)_4_	−9.71	−9.24	−4.56	−4.87	−7.59	6.48 [28]
B(CN)_4_	−8.65	−9.01	−5.18	−5.32	−8.33	6.30 [29,30]
BETI	−8.49	−8.24	−3.86	−4.26	−6.99	5.65 [31]
BF_4_	−9.64	−9.55	−3.13	−3.76	−6.82	5.76 [24,30,32,33,34]
BH_4_	−4.67	−4.67	−0.87	−0.85	−3.72	2.0 [35]
BOB	−8.61	−8.32	−3.7	−4.06	−6.81	4.5 [36]
Cl	−3.38	−3.38	0.4	0.27	−2.5	3.60 [24,32,34,37]
ClO_4_	−7.33	−7.21	−2.14	−2.63	−5.48	5.71 [24,38]
cyanopyrrolide	−2.81	−2.77	−0.49	−0.53	−3.06	3.37 [39]
DCA	−4.32	−4.31	−0.97	−1.1	−3.74	2.82 [24,40]
FAP	−8.83	−8.38	−4.08	−4.39	−7.00	5.72 [29,32]
FSI	−8.34	−8.16	−3.72	−4.06	−6.85	5.62 [40,41]
FTFSI	−8.2	−7.93	−3.63	−3.97	−6.71	5.00 [42]
HSO_4_	−6.48	−6.24	−1.56	−1.88	−4.81	6.86 [24]
IM1_4_	−8.47	−8.22	−3.84	−4.24	−6.97	4.85 [31,43]
Im(BF_3_)_2_	−5.81	−5.99	−3.3	−3.33	−6.11	5.35 [44]
NO_3_	−5.72	−5.39	0.01	−0.63	−3.48	4.57 [24]
ODFB	−7.73	−7.41	−3.98	−3.1	−5.86	4.2 [45]
PF_6_	−10.86	−10.71	−4.27	−4.92	−7.94	5.38 [32,34]
TFO	−7.01	−6.84	−2.02	−2.47	−5.33	5.50 [24,32,34]
TFSAM	−6.11	−6.16	−2.43	−2.67	−5.38	5.70 [40]
TFSI	−8.32	−8.06	−3.67	−4.07	−6.8	5.65 [24,29,32,34,40]
thiocyanate	−3.42	−3.44	−0.41	−0.4	−3.08	3.65 [46,47]
triazolide	−3.18	−3.31	−0.54	−0.6	−3.38	3.57 [39]
TSAC	−6.85	−6.72	−2.81	−3.11	−5.81	6.02 [40]

**Table 2:** Mean Squared Error (MSE) values obtained for the comparison between the experimental values of the anodic limit and those calculated by various functionals.

Functional	Type of Approximation	MSE (V^2^)
M08-HX	GH-mGGA	1.61
M11	RSH-mGGA	1.64
M06-2X	GH-mGGA	1.81
MN-12SX	RSH-mGGA	1.81
MP2	--	1.87
M11-L	mGGA	1.97
BMK	mGGA	2.00
ωB97X-D	RSH-GGA	2.18
ωB97M-V	RSH-mGGA	2.22
CAM-B3LYP	RSH-GGA	2.27
B3LYP	GH-GGA	2.78

## Data Availability

Data are contained within the article or Supplementary Materials.

## Supplementary Materials

The following supporting information can be downloaded at: https://www.mdpi.com/article/10.3390/e25050793/s1. Figure S1: Experimental anodic limit versus the energy (in atomic units), LUMO and HOMO levels (in eV) and the minimum of the ionization potential (in eV) calculated at the Hartree–Fock level using the 6-31G** basis set. The red curves are the best-fit lines obtained by a linear regression. Figure S2: Experimental anodic limit versus the anodic limit calculated by means of the empirical model proposed in the text. The red curve is the best-fit line obtained by a linear regression.
