# Supplementary material for: Some Considerations about the Anodic Limit of Ionic Liquids Obtained by Means of DFT Calculations
Source: Entropy (Basel). 2023 May 12;25(5):793. doi: 10.3390/e25050793 (PMC10217212; doi:10.3390/e25050793)
Supplement: Supplementary file 1 [file entropy-25-00793-s001.zip › entropy-2355626-supplementary.pdf]

entropy

MDPI

Supporting Information

Some Considerations about the Anodic Limit of Ionic Liquids Obtained by Means of DFT Calculations

Annalisa Paolone [1,*], Simone Di Muzio [1,2], Oriele Palumbo [1] and Sergio Brutti [1,3]

1 Consiglio Nazionale delle Ricerche, Istituto dei Sistemi Complessi, Piazzale Aldo Moro 5, 00185 Rome, Italy; simone.dimuzio1@graduate.univaq.it (S.D.M.); oriele.palumbo@roma1.infn.it (O.P.); sergio.brutti@uniroma1.it (S.B.)
2 Department of Physical and Chemical Sciences, University of L'Aquila, Via Vetoio, 67100 L'Aquila, Italy
3 Department of Chemistry, Sapienza University of Rome, Piazzale Aldo Moro 5, 00185 Rome, Italy
* Correspondence: annalisa.paolone@roma1.infn.it

Citation: Paolone, A.; Di Muzio, S.; Palumbo, O.; Brutti, S. Some Considerations about the Anodic Limit of Ionic Liquids Obtained by Means of DFT Calculations. *Entropy* **2023**, *25*, 793. https://doi.org/10.3390/e25050793

Academic Editor: Antonio M. Scarfone

Received: 4 April 2023
Revised: 5 May 2023
Accepted: 11 May 2023
Published: 12 May 2023

Copyright: © 2023 by the authors. Licensee MDPI, Basel, Switzerland. This article is an open access article distributed under the terms and conditions of the Creative Commons Attribution (CC BY) license (https://creativecommons.org/licenses/by/4.0/).

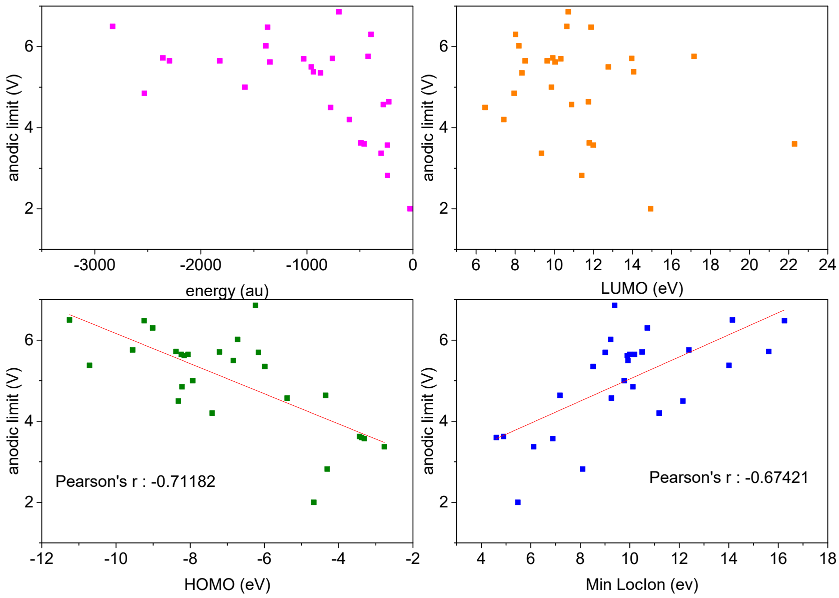

Figure S1. Experimental anodic limit versus the energy (in atomic units), LUMO and HOMO levels (in eV) and the minimum of the ioniziation potential (in eV) calculated at the Hartree-Fock level using the 6-31G** basis set. The red curves are the best fit lines obtained by a linear regression.

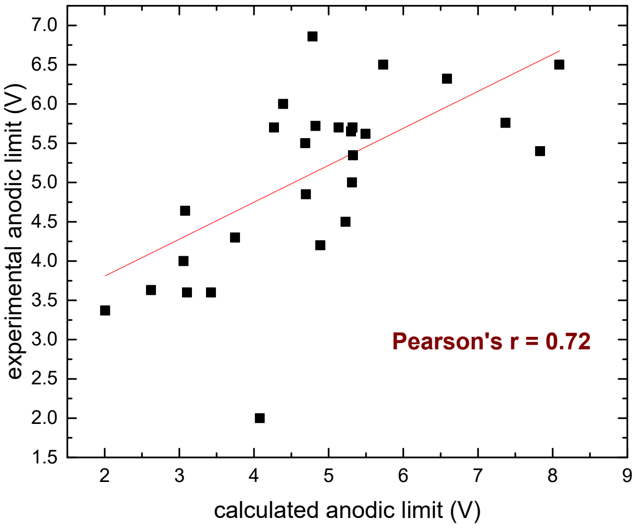

Figure S2. Experimental anodic limit versus the anodic limit calculated by means of the empirical model proposed in the text. The red curve is the best fit line obtained by a linear regression.
